# Transcriptome analysis of the epidermis of the *purple quail-like* (*q-l^p^*) mutant of silkworm, *Bombyx mori*

**DOI:** 10.1371/journal.pone.0175994

**Published:** 2017-04-17

**Authors:** Pingyang Wang, Zhiyong Qiu, Dingguo Xia, Shunming Tang, Xingjia Shen, Qiaoling Zhao

**Affiliations:** 1 School of Biotechnology, Jiangsu University of Science and Technology, Zhenjiang, Jiangsu, China; 2 The Sericulture Research Institute, Chinese Academy of Agricultural Sciences, Zhenjiang, Jiangsu, China; Institute of Plant Physiology and Ecology Shanghai Institutes for Biological Sciences, CHINA

## Abstract

A new *purple quail-like* (*q-l*^*p*^) mutant found from the plain silkworm strain 932VR has pigment dots on the epidermis similar to the pigment mutant *quail* (*q*). In addition, *q-l*^*p*^ mutant larvae are inactive, consume little and grow slowly, with a high death rate and other developmental abnormalities. Pigmentation of the silkworm epidermis consists of melanin, ommochrome and pteridine. Silkworm development is regulated by ecdysone and juvenile hormone. In this study, we performed RNA-Seq on the epidermis of the *q-l*^*p*^ mutant in the 4^th^ instar during molting, with 932VR serving as the control. The results showed 515 differentially expressed genes, of which 234 were upregulated and 281 downregulated in *q-l*^*p*^. BLASTGO analysis indicated that the downregulated genes mainly encode protein-binding proteins, membrane components, oxidation/reduction enzymes, and proteolytic enzymes, whereas the upregulated genes largely encode cuticle structural constituents, membrane components, transport related proteins, and protein-binding proteins. Quantitative reverse transcription PCR was used to verify the accuracy of the RNA-Seq data, focusing on key genes for biosynthesis of the three pigments and chitin as well as genes encoding cuticular proteins and several related nuclear receptors, which are thought to play key roles in the *q-l*^*p*^ mutant. We drew three conclusions based on the results: 1) melanin, ommochrome and pteridine pigments are all increased in the *q-l*^*p*^ mutant; 2) more cuticle proteins are expressed in *q-l*^*p*^ than in 932VR, and the number of upregulated cuticular genes is significantly greater than downregulated genes; 3) the downstream pathway regulated by ecdysone is blocked in the *q-l*^*p*^ mutant. Our research findings lay the foundation for further research on the developmental changes responsible for the *q-l*^*p*^ mutant.

## Introduction

Almost all insects are pigment, and these colorful markings play important roles in many physiological and biochemical aspects of mimicry, damage avoidance, mate attraction, thermoregulation, immunity, and cuticle hardening [1–6]. Pigmentation is an important biological characteristic in insects. There are three main types of pigments melanin, ommochromes and pteridines [7, 8], among which melanin research has been relatively extensive in *Drosophila* [4, 9–14]. The conversion of tyrosine into dopa is based on the action of tyrosine hydroxylase (*TH*), and partial dopa is transformed into dopamine via dopa decarboxylase (*Ddc*). The *ebony* gene encodes N-β-alanyl-dopamine synthase, which has functions in the process of dopamine conversion to N-β-alanyl-dopamine (NBAD) coupled with β-alanine. Dopamine can also be transformed into N-acetyl dopamine (NADA) via dopamine acetyltransferase (*Dat 1*). NADA and NBAD are then oxidized into NADA sclerotin (colorless) and NBAD sclerotin (yellowish), respectively. Dopa and dopamine can be converted into dopa melanin and dopamine melanin, respectively, via the products of the *yellow*, *yellow-f*, *yellow-f2* and *phenol oxidase* genes. Homologous genes have been found in *Bombyx mori*, and alterations in the *ebony* and *yellow* genes are responsible for the chocolate silkworm mutant (*ch*) and sable silkworm mutant (*so*), respectively [15]. The sex-linked chocolate silkworm mutant (*sch*) is caused by a mutation of the control region of the gene encoding tyrosine hydroxylase (*TH*) [16].

The study of pigmentation in silkworms is largely based on various mutants. At present, pigmentation mutants have been identified during all developmental stages, including eggs, larvae, pupae and adults. The normal color of silkworm eggs is brown or black; in contrast, a white egg mutant (*w-2*) characterized by white eggs is caused by mutation in a gene that encodes a transport protein of ommochromes and leads to a lack of ommochromes in the oolemma [17]. Pigmentation mutants are more abundant in larvae than in other stages. The genes of several chocolate silkworm mutants (*Ia*, *ch*, *ch*^*p*^, *ch-2*, *cm*, and *sch*) are located on different chromosomes, indicating that they have different regulation mechanisms despite similar phenotypes. The *mln* mutant is characterized by body surface blackening in larvae and adults; it is caused by mutation of the *BM-iAANAT* gene, which is homologous to *Dat1* in the melanin synthesis pathway in *Drosophila*. A partial sequence in the 4^th^ exon of *BM-iAANAT* is lost, and defunctionalization of N-acetyl-dopamine transferase leads to accumulation of dopamine, blackening the body surface [18, 19]. The *so* mutant, a typical pupa color mutant, is caused by a mutation in *ebony*, with both larvae and pupae being characterized by a black surface [15].

**Fig 1:**
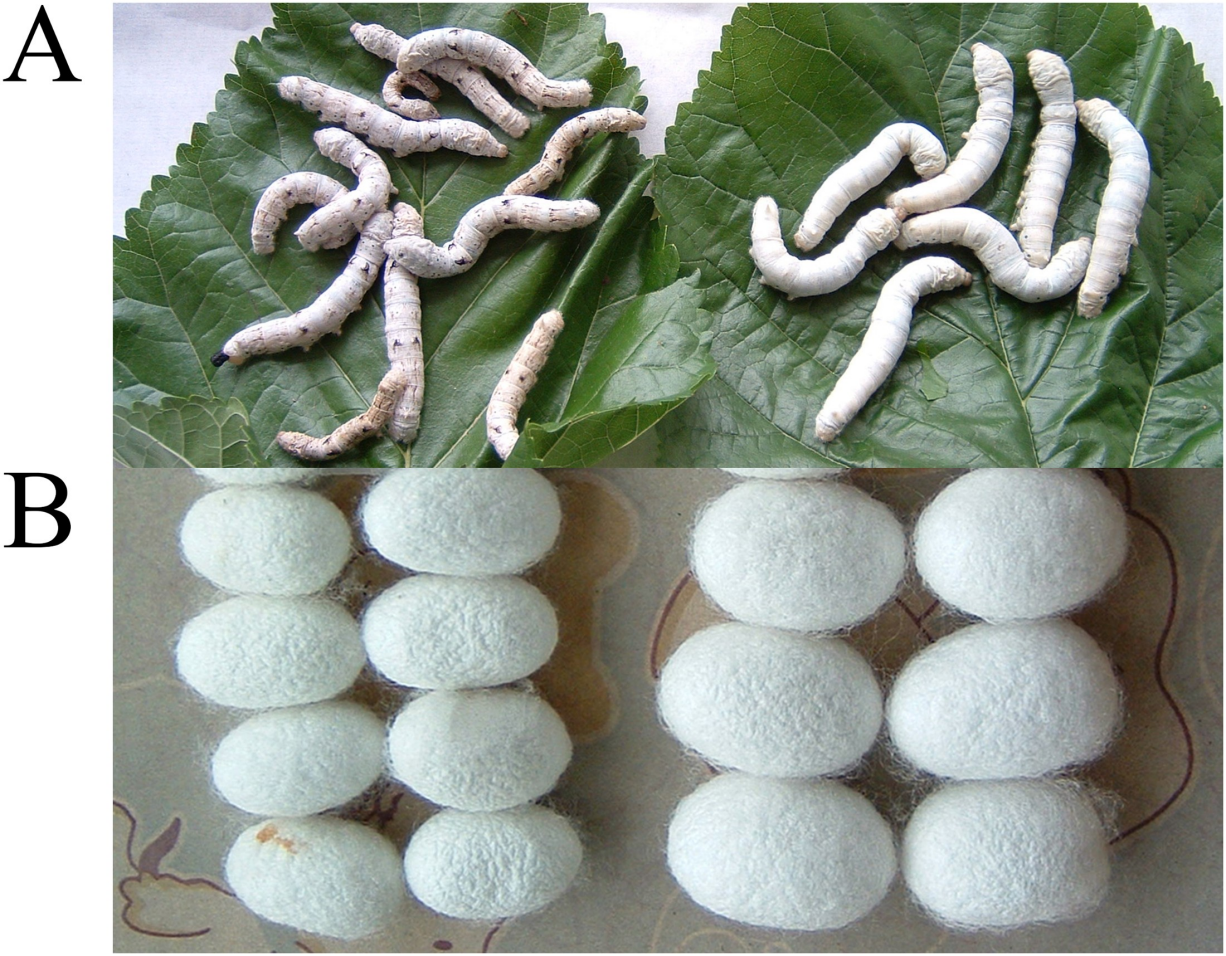
The phenotype of wild-type 932VR and mutant *q-l*^*p*^. (A) The left panel shows the mutant *q-l*^*p*^ silkworm, and the right panel shows the wild-type 932VR silkworm. (B) The left panel shows the mutant *q-l*^*p*^ silkworm cocoon, and the right panel shows the wild-type 932VR silkworm cocoon.

The mutant purple quail-like (*q-l*^*p*^) is a new pigment mutant (Fig 1) obtained from the plain silkworm strain 932VR. The characteristics of *q-l*^*p*^ are inherited stably after more than 10 generations of inbreeding. Compared with individual exhibiting normal markings, the *q-l*^*p*^ mutant has no obvious eye-spots but normal star-spots and semilunar marking. There are dots and lines with longitudinal wave markings on the dorsal sides of the 6^th^ to 7^th^ abdominal segments, which constitute quail markings, in between the star-spots and semilunar markings. The whole-body markings are very similar to those of the *quail* mutant (*q*) [20, 21]. The larvae of *q-l*^*p*^ show light purple skin, eat only small amounts of mulberry leaves, are weak, and develop slowly and unevenly; in addition their larval bodies and cocoons are small [21] (Fig 1). Although the phenotype of the mutant is very similar to *quail*, genetic analysis indicates that they are not controlled by the same gene. The genes mutated in *q-l*^*p*^ and *q* are located on the 8^th^ chromosome and 7^th^ chromosome, respectively [21]. Previous work indicated that synthesis of melanin, ommochrome and pteridine are all increased in quail [22, 23]. In this study, the epidermises of *q-l*^*p*^ approximately 6 hours after molting were used as materials for RNA-Seq; the epidermises of 932VR approximately 6 hours after molting served as the control. Biosynthetic genes for the three types of pigments and chitin and those encoding cuticle proteins and nuclear receptors were analyzed between 932VR and *q-l*^*p*^. The results lay a foundation for further research of the *q-l*^*p*^ formation mechanism.

## Materials and methods

### The silkworm strains

The silkworm strains 932VR and *q-l*^*p*^ were provided by The Sericulture Research Institute, Chinese Academy of Agricultural Sciences (Zhenjiang, China). The silkworms were fed fresh mulberry leaves under standard conditions with alternating 12 hours of illumination and 12 hours of darkness at 25±2°C. Epidermis samples were collected from every three silkworms after 6 hours of molting in the 4^th^ instar. All samples were frozen and stored at -80°C.

### RNA extraction and transcriptome sequencing

Total RNA was extracted using RNAiso Plus (TaKaRa, China) and dissolved in RNase-free water. The concentration of total RNA was determined using a NANODROP1000 microspectrophotometer (Thermo, USA) after treatment with DNase. Total mRNA was enriched using Oligo (dT) magnetic beads, after which the mRNAs were sheared into short fragments using hybridization-interruption reagents. These short RNA fragments were then synthesized into double-stranded cDNA using six-base random primers, and terminal modification was performed for the purified double-stranded cDNA; a base (A) tail was added, and the fragments were ligated. The quality of the cDNA library constructed was tested using an Agilent 2100 Bioanalyzer; after quality criteria were met, sequencing was executed using an Illumina HiSeqTM2500 system (Illumina, USA).

### RNA-Seq data analysis

Quality analysis was conducted for the original data obtained using the Illumina HiSeqTM2500 system; after quality criteria were met, clean reads were screened by filtering out low-quality reads. The sequences were aligned to the silkworm genome database SilkDB (http://silkworm.swu.edu.cn/silkdb/); after a second quality analysis for alignment, analysis of the distribution and coverage of the clean reads on the reference sequence was conducted. RPKM (Reads Per Kb per Million reads) [24] was used to calculate the expression level of genes, with RPKM = mapped reads of gene/(the total mapped reads of all genes*the length of this gene)*10^9. The RPKM of a gene ranged up to 5, and difference in expression was considered at P < 0.05; the fold change of *q-l*^*p*^ and 932VR RPKMs was greater than 2. The function of the differentially expressed genes and pathways related to pigment were analyzed using BLASTGO and KEGG, respectively.

### Quantitative reverse transcription PCR

Epidermis samples were collected from every three silkworms after 6 hours of molting in the 4^th^ instar, and total RNA was prepared using RNAiso Plus and reverse transcribed using the Reverse Transcriptase M-MLV (RNase H-) kit (TaKaRa, China) after treatment with DNase. The cDNA was diluted to 20 ng/μL and used as the template for qRT-PCR. The 20-μL reaction included 1 μL primer (10 μmol/L, S1 Table), 1 μL cDNA, 10 μL 2×SYBR^®^ Premix Ex Taq^™^ (Tli RNaseH Plus) (TaKaRa, China) and 8 μL ddH_2_O. After a rapid centrifugation step, quantitative reverse transcription PCR (qRT-PCR) was performed using a LightCycle 96 real time PCR system (Roche, Switzerland) with the reaction program below: a three-step reaction protocol of 45 cycles of 95°C for 10 s, 58°C for 10 s and 72°C for 10 s after a 10-min step of pre-degeneration, followed by melting. Relative expression was calculated using the 2^–ΔΔCt^ method [25] with glyceraldehyde-3-phosphate dehydrogenase (*GAPDH*, *BGIBMGA007490*) as the reference gene. Melting analysis was performed to guarantee the qRT-PCR quality. The relative expression of 932VR and *q-l*^*p*^ were compared, and RNA-Seq and qRT-PCR data were compared to analyze several key pathways.

## Results and discussion

### General information of RNA-Seq

**Fig 2:**
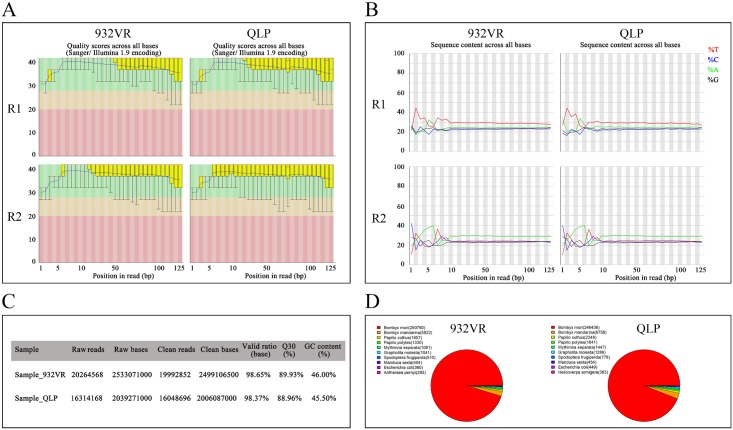
Quality evaluation of the RNA-Seq data. (A) Quality evaluation of bases. (B) Base distribution. (C) Pretreatment of the data quality. (D) Pollution detection of reads.

Prior to bioinformatic analysis, we assessed the quality of the transcriptome sequencing data, including quality evaluation of bases, base distribution, pretreatment of the data quality and pollution detection of reads (Fig 2). As expected, all of test results met the requirements. For 932VR, we obtained 19,992,852 paired-end reads, of which 13,792,700 reads were aligned to the silkworm genome database (68.99%) and 11,357,633 to silkworm genes (56.80%). For *q-l*^*p*^, we obtained 16,048,696 paired-end reads, of which 10,987,505 reads were aligned to the silkworm genome database (68.46%) and 8,639,063 to silkworm genes (53.83%). Expression of 14,623 silkworm genes was calculated based on the RPKM method (S2 Table), where an RPKM for one gene less than 5 was regarded as scarcely expressed. At a P value < 0.05, a difference in expression was considered to exist when the ratio of the RPKM for *q-l*^*p*^ and 932VR was up to 2 or less than 0.5. The results indicated 515 differentially expressed genes between 932VR and *q-l*^*p*^, of which 234 were upregulated and 281 downregulated (S3 Table).

**Fig 3:**
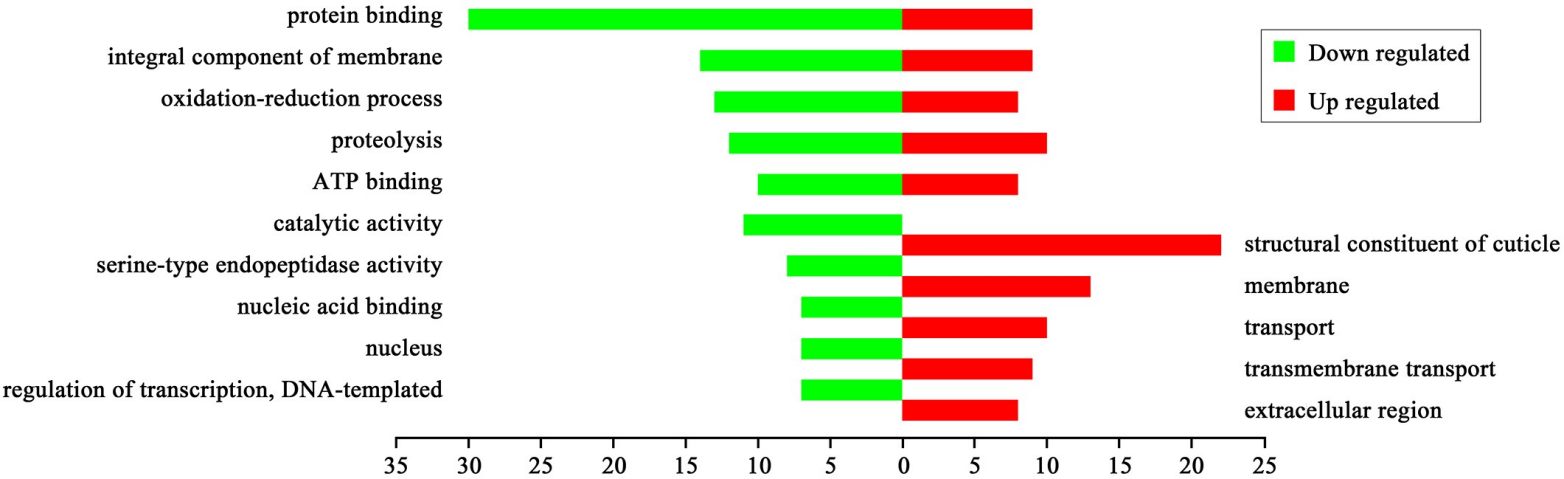
Functional categories of differentially expressed genes in the *q-l*^*p*^ mutant.

Based on BLASTGO analysis, we examined the top 10 upregulated and downregulated genes in *q-l*^*p*^ compared to 932VR. The upregulated genes mainly encode structural constituents of the cuticle, membrane proteins, transport related proteins, protein binding proteins, enzymes catalyzing oxidation-reduction process, hydrolases, and ATP-binding proteins. The downregulated genes mainly encode protein-binding proteins, structural constituents of the membrane, transport related proteins, enzymes catalyzing oxidation-reduction process, hydrolases, ATP-binding proteins, nucleic acid-binding proteins, and transcription factors. (Fig 3).

### Verifying the accuracy of the RNA-Seq data by qRT-PCR

First, we analyzed several housekeeping genes. Although no significant difference between the wild-type 932VR and the mutant *q-l*^*p*^ was observed for glyceraldehyde-3-phosphate dehydrogenase (*BGIBMGA007490*, fold = 1.34) or ribosomal protein L3 (*BGIBMGA013567*, fold = 1.05), a significant difference for BmActin3 (*BGIBMGA005576*, fold = 0.26) was found. We then determined that the accuracy of the RNA-Seq data reached the required level. Based on manifestations of the mutants and the differentially expressed genes according to RNA-Seq, we selected certain genes for qRT-PCR testing, including differentially expressed genes, key biosynthesis genes for the three types of pigments and chitin and genes coding cuticular proteins and several related nuclear receptors. The results are listed in Table 1 and S1 Table.

**Table 1:** Comparison of RPKM between qRT-PCR.

Team	Gene name	Gene id.	Fold change (QLP/932VR)		Consistency	
			RPKM	qRT-PCR	Up	Down
(a)	larval cuticle protein LCP-22	BGIBMGA000332	47.93	7.14	√	
	larval cuticle protein LCP-12	BGIBMGA002548	29.01	1.59	√	
	cuticular protein	BGIBMGA003728	52.36	0.11	×	
	cuticular protein	BGIBMGA005303	0.01	0.04		√
	myosin heavy chain, non-muscle	BGIBMGA005536	0.00	0.08		√
	alanine-tRNA ligase, cytoplasmic	BGIBMGA006216	266	0.10	×	
	cuticular protein glycine-rich	BGIBMGA008212	134.98	1.12	√	
	unknown	BGIBMGA009159	0.02	0.04		√
	cytochrome P450	BGIBMGA009522	0.00	0.01		√
	nesprin-1	BGIBMGA010472	0.02	0.07		√
	trypsin-1	BGIBMGA013698	Inf	1.40	√	
	mucin	BGIBMGA013949	0.03	0.08		√
(b)	N-β-alanyl-dopamine synthase	BGIBMGA000031	Ne	Ne	Ne	Ne
	tyrosine hydroxylase	BGIBMGA000563	1.41	0.63	×	
	yellow	BGIBMGA001149	55.33	0.02	×	
	N-β-alanyl-dopamine hydrolase	BGIBMGA002077	1.41	0.00	×	
	dopa decarboxylase	BGIBMGA003199	1.13	19.38	√	
	phenylalanine hydroxylase	BGIBMGA003866	0.39	0.77		√
	yellow-f4-2	BGIBMGA003918	Ne	Ne	Ne	Ne
	yellow-e	BGIBMGA007253	0.51	0.05		√
	yellow-h2	BGIBMGA007255	22.30	17.47	√	
	dopamine acetyltransferase	BGIBMGA008538	3.99	10.46	√	
	blank	BGIBMGA012088	Inf	47.40	√	
	yellow-f2	BGIBMGA014032	0.28	0.21		√
	yellow-x	BGIBMGA014224	0.75	0.32		√
(c)	phenoxazinone synthetase	BGIBMGA006740	1.26	2.85	√	
	ommochrome-binding protein 1	BGIBMGA007285	0.46	1.57		×
	ommochrome-binding protein 2	BGIBMGA007286	0.42	0.96		√
	kynurenine formamidase	BGIBMGA007856	1.91	1.68	√	
(d)	GTP- cyclohydrolase -a	BGIBMGA001235	0.72	0.78		√
	GTP- cyclohydrolase -b	BGIBMGA008134	1.41	39.31	√	
(e)	FTZ-F1	BGIBMGA000716	2.04	2.82	√	
	HR38	BGIBMGA002964	1.54	2.51	√	
	ecdysone receptor	BGIBMGA006767	0.72	0.52		√
	HR39	BGIBMGA007914	3.22	3.87	√	
	E74a	BGIBMGA007970	0.87	5.34		×
(f)	UDP-N-acetylglucosamine pyrophosphorylase	BGIBMGA001609	1.36	2.75	√	
	glucose-6-phosphate isomerase	BGIBMGA004221	0.95	0.19		√
	glutamine: fructose-6-phosphate-aminotransferase	BGIBMGA007517	3.71	3.67	√	
	trehalase	BGIBMGA005665	2.16	5.71	√	
	glucosamine-6-phosphate N-acetyltransferase	BGIBMGA005161	0.64	6.76		×
	β-N-acetyl-glucosaminidase	BGIBMGA011646	0.61	0.24		√
	chitinase	BGIBMGA008709	0.17	0.00		√
	chitinase	BGIBMGA010240	0.08	0.00		√
(g)	BmActin3	BGIBMGA005576	0.26	0.22		√

(a) prominently differentially expressed genes among RNA-seq data; (b) key genes for biosynthesis of melanin; (c) key genes for biosynthesis of ommochrome; (d) key genes for biosynthesis of pteridine; (e) Genes encoding several related nuclear receptors; (f) key genes for biosynthesis of chitin; (g) BmActin3

**Inf**: gene expressed in *q-l*^*p*^ but not in 932VR

**Ne**: not expressed in neither *q-l*^*p*^ nor 932VR

### Differentially expressed genes

After analysis of differentially expressed genes revealed by RNA-Seq, we selected 12 strongly differentially expressed genes for qRT-PCR testing; the results are listed in Table 1. Of the 12 genes, 6 were upregulated by RPKM and 6 downregulated. qRT-PCR results showed that 10 genes were consistent with the RPKM values. The remaining two genes were upregulated according to RPKM but downregulated according to qRT-PCR. These results indicated an effective rate of RNA-Seq of 83.33% and that the RNA-Seq data were reliable. Among the 10 genes strongly differentially expressed between 932VR and *q-l*^*p*^, four appear to encode cuticle proteins, one encodes the heavy chain of myosin, one encodes trypsin 1, one encodes nesprin 1, one encodes mucin, one encodes a member of the cytochrome P450 family and one is unknown.

The main phenotype of *q-l*^*p*^ is an epidermis that is similar in appearance to the pigment mutant *quail*; the pigment is one of the important characteristics of the epidermis [26], and there are many cuticle proteins in the epidermis [27]. Indeed, almost half of the 10 strongly differentially expressed genes encode cuticle proteins, and they may play key roles in formation of the epidermis and pigmentation. Members of the cytochrome P450 family comprise monooxygenases belonging to the CYP gene family [28]. In insects, cytochrome P450 is involved in many anabolism processes, including metabolism of exogenous substances such as plant secondary metabolites and pigments, as well as endogenous substances such as juvenile hormone, ecdysone, fatty acid, and pheromones [29]. Downregulation of cytochrome P450 in *q-l*^*p*^ may influence the synthesis and signaling of a series of hormones. Trypsin is a protein hydrolase, a large class of differentially expressed genes in *q-l*^*p*^. Mucin, mainly distributed in epithelial cells, has a protective role inside cells and can also contribute to a protective extracellular mucin gel [30]. Downregulation of mucin in *q-l*^*p*^ may be related to its high death rate, as *q-l*^*p*^ is more easily damaged by adverse environmental factors. Nesprin 1, a component of the karyotheca, belongs to a nuclear skeleton and cytoskeleton protein family that contains multiple repetitive sequences, the function of which is to connect the cell nucleus to the actin cytoskeleton, affecting myokinesis [31]. The heavy chain of myosin (fold = 0.42) and BmActin3 (fold = 0.26) were downregulated, similar to nesprin 1 (fold = 0.05). Such genes are directly related to muscle contraction [32], and their downregulation might influence mobility and vitality, consistent with the *q-l*^*p*^ larval phenotype of reduced food consumption, slow growth and low vitality.

### Differentially expressed pigment biosynthesis genes

**Fig 4:**
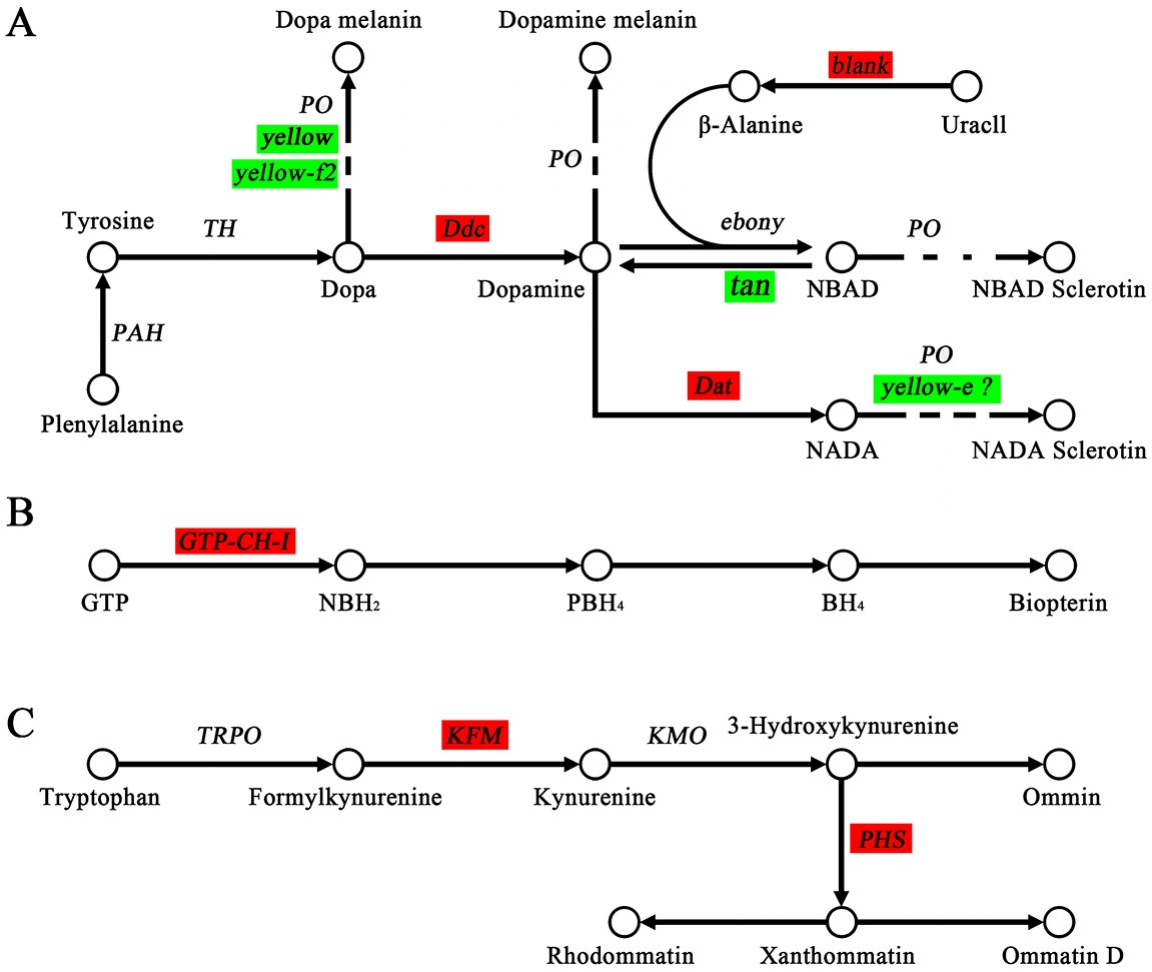
The pigment pathways of melanin ^A^, pteridine ^B^ and ommochrome ^C^. Genes with green background represent their downregulation and red for upregulation in *q-l*^*p*^.

Melanin is one of the most important pigments, and it has been studied in detail in *Drosophila melanogaster* [4, 9–14]. We analyzed key genes of melanin biosynthesis based on the synthesis pathway of *D*. *melanogaster* (Fig 4A). Melanin is produced from tyrosine by a series of enzymatic reactions. Tyrosine is produced from phenylalanine via phenylalanine hydroxylase (*PAH*) [33] and is then transformed to dopa via tyrosine hydroxylase (*TH*). The results for *PAH* (fold = 0.77) and *TH* (fold = 0.63) expression in *q-l*^*p*^ were not obviously different from the results for 932VR. However, *yellow* (fold = 0.02) and *yellow-f2* (fold = 0.21) were downregulated in *q-l*^*p*^; these two genes play key roles in the processing of dopa to dopa melanin [12, 15]. Dopa decarboxylase (*Ddc*, fold = 19.38) catalyzes the step from dopa to dopamine, and dopamine acetyltransferase (*dat*, fold = 10.46) catalyzes the step from dopamine to N-acetyl dopamine (NADA); both were upregulated in *q-l*^*p*^, leading to accumulation of dopamine and NADA. *Yellow-e* catalyzes the reaction from NADA to NADA sclerotin, and downregulation of *yellow-e* (fold = 0.05) leads to further accumulation of dopamine and NADA. Silencing *ebony*, which encodes the enzyme catalyzing the reaction from dopamine to N-β-acetyl dopamine (NBAD), also leads to further accumulation of dopamine and NADA. A lack of *ebony* might be a reason for the downregulation of *tan* (fold = 0), which catalyzes the step from NBAD to dopamine and the upregulation of *blank* (fold = 47.4), which catalyzes the step from uracil to β-alanine [34]. Upregulation of *Ddc* leads to most available dopa being transformed into dopamine, and a decrease in dopa and downregulation of *yellow* and *yellow-f2* lead to a lack of dopa melanin. However, accumulation of dopamine causes production of more dopamine melanin, resulting in brownness, and might be responsible for the mutant *q-l*^*p*^.

GTP cyclohydrolase (*GTP-CH*) is the first enzyme in the biosynthesis of pteridine [20, 35]. Two types of *GTP-CH*s are expressed differentially; no difference in *GTP-CH I a* (fold = 0.78) was observed between 932VR and *q-l*^*p*^, though *GTP-CH I b* (fold = 39.31) was markedly upregulated in *q-l*^*p*^, which is consistent with *quail* [20]. Upregulation of *GTP-CH I* would cause an increase in BH2, followed by an increase in BH4, and eventually cause an increase in biological pteridine, which might lead to pigment deposition in the epidermis (Fig 4B).

Ommochrome is a metabolite of tryptophan and can be transformed into different colors varying from red, purple, black to yellow by participating in redox reactions [36]. As an excess of tryptophan is poisonous to an organism, transforming tryptophan to ommochrome avoids tryptophan accumulation [7]. Tryptophan 2,3-dioxygenase plays a role in converting tryptophan to formylkynurenine. Kynurenine is then produced from formylkynurenine via kynurenine formamidase (*KFM*) and converted to 3-hydroxy kynurenine. Xanthommatin is a ommochrome derived from two molecules of 3-hydroxy kynurenine through the action of phenoxazinone synthetase (*PHS*) [37, 38]. In *q-l*^*p*^, these two key genes, *KFM* (fold = 1.68) and *PHS* (fold = 2.85), were upregulated, which might result in more xanthommatin production. Ommochrome-binding protein (*OBP*) is a key pigmentation-related protein in the epidermis [39]. According to our RNA-Seq results, two *OBP*s are downregulated in *q-l*^*p*^. However, after confirmation using qRT-PCT, *OBP1* (fold = 1.57) was shown to be upregulated in *q-l*^*p*^, with no difference in *OBP2* (fold = 0.96) between 932VR and *q-l*^*p*^. These results also correspond to the *quail* mutant [20]. Upregulation of *KFM* and *PHS* leads to accumulation of ommochrome, and upregulation of OBP enhances this process, which might facilitate the observed pigmentation in *q-l*^*p*^ (Fig 4C).

### Differentially expressed genes encoding cuticle proteins

**Fig 5:**
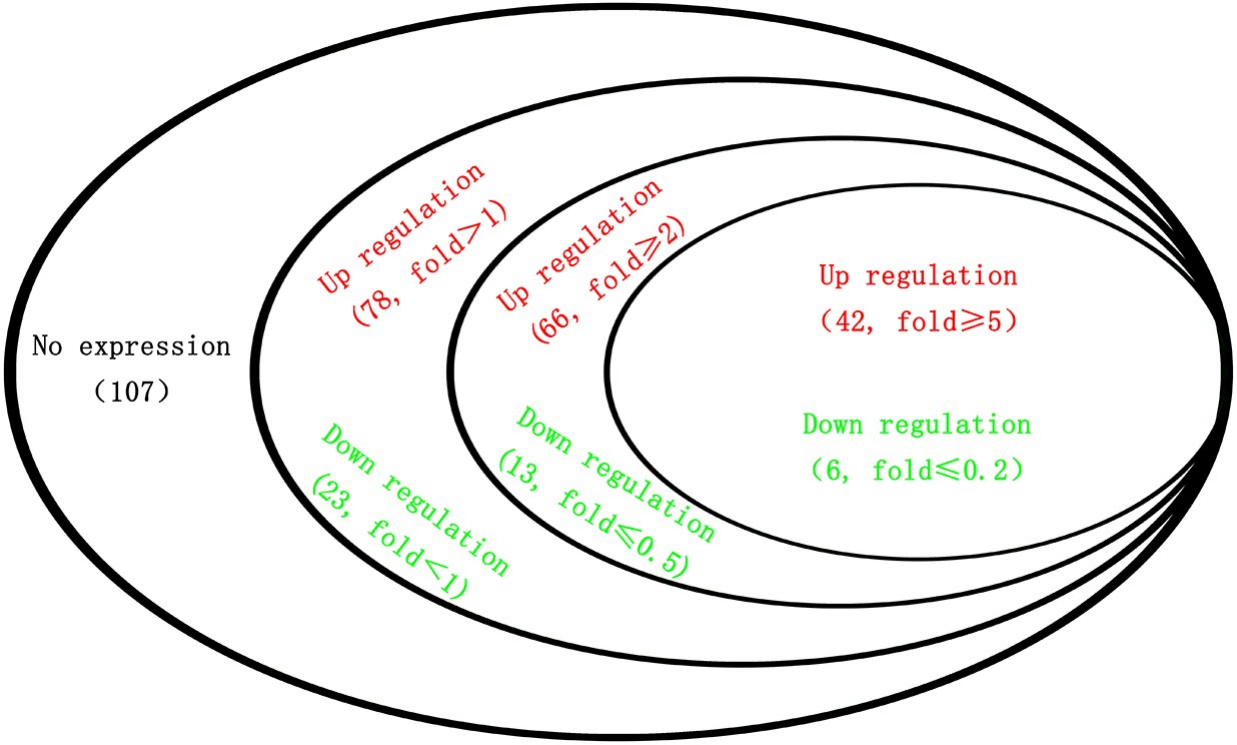
Differentially expressed cuticular genes.

The epidermis is a very important organ of the silkworm, providing protection for external adverse environmental factors. The silkworm must go through several molting processes in larval development stages, during which, the old epidermis is replaced by a new epidermis. To maintain epidermal markings, pigment is deposited in every molt. The epidermis is composed of a large number of cuticle proteins and chitin. There are more than 200 cuticle protein genes in *B*. *mori* [27] (S4 Table), of which 107 were not expressed (RPKM<5 in both 932VR and *q-l*^*p*^) and 101 were expressed. Of the 101 expressed genes, 78 were upregulated and 23 downregulated in *q-l*^*p*^. In addition, 66 and 13 genes were upregulated and downregulated ≥2-fold in *q-l*^*p*^; for fold changes ≥5 in *q-l*^*p*^, we found 42 upregulated genes and 6 downregulated genes (Fig 5).

The results above reveal more upregulated genes (77.2%) than downregulated genes (22.8%), and for fold changes ≥5, the ratio of upregulated genes reached 87.5%. In *q*, the inverse is true: of 62 differentially expressed genes, only 15 genes are upregulated and 47 downregulated, with a ratio of upregulated genes of 24.2% [20]. By comparing samples, we found that the control *q* strain Dazao exhibits normal markings but that the control *q-l*^*p*^ 932VR strain is a plain silkworm. Therefore, some of these upregulated genes might be associated with normal marking formation.

By comparing differentially expressed cuticular genes between *q*/Dazao and *q-l*^*p*^/932VR, we found 5 upregulated genes and 3 downregulated genes in *q* and *q-l*^*p*^ as fold≤0.5 or fold≥2 (Table 2). These genes might play key roles in the pigmentation of *q* and *q-l*^*p*^.

**Table 2:** Comparison of cuticular genes between *q*/Dazao and *q-l*^*p*^ /932VR.

Gene id.	Description	Fold change		Up or Down	
		q/Dazao	*q-l*^*p*^ /932VR		
BGIBMGA000274	RR2	0.15	0.00		Down
BGIBMGA000371	RR1	4.29	2.35	Up	
BGIBMGA001444	RR1	0.31	0.12		Down
BGIBMGA002384	glycine-rich	3.67	5.85	Up	
BGIBMGA002385	glycine-rich	2.25	3.35	Up	
BGIBMGA002549	RR1	2.54	5.29	Up	
BGIBMGA008254	hypothetical	2.04	Inf	Up	
BGIBMGA012213	RR1	0.32	0.00		Down

**Inf**: the gene is expressed in *q-l*^*p*^ but not in 932VR

### Differentially expressed genes in chitin synthesis

Moreover, we analyzed several chitin metabolism and biosynthesis related enzymes. Chitin is widely distributed in nature, and the main component is glycosaminoglycan [40], which constitutes the insect cuticle, trachea, and peritrophic membrane of the digestive tube, by binding proteins to protect the insect from damage [41]. The synthesis of chitin begins with glucose, with eventual conversion to UDP-N-acetylglucosamine [42]. There are many enzymes in the pathway, including trehalase, hexokinase, glucose-6-phosphate isomerase, glutamine:fructose-6-phosphateaminotransferase, glucosamine-6-phosphate-N-acetyltransferase, phosphoacetyl-glucosamine mutase, UDP-N-acetylglucosamine pyrophosphorylase and chitin synthetase [43].

Chitin synthesis is a complex process. We chose five key enzymes for analysis: trehalase, glucose-6-phosphate isomerase (G6PI), glutamine: fructose-6-phosphate-aminotransferase (GFPA), glucosamine-6-phosphate N-acetyltransferase (G6PNA) and UDP-N-acetylglucosamine pyrophosphorylase (UNAP), of which UNAP is the rate-limiting enzyme [43]. The results indicated G6PI (fold = 0.19) to be downregulated and trehalase (fold = 5.71), GFPA (fold = 3.67), G6PNA (fold = 6.76) and UNAP (fold = 2.75) upregulated in *q-l*^*p*^, which might lead to enhanced chitin synthesis. In the chitin metabolism pathway, after chitin is degraded to oligomers via chitinase, β-N-acetyl-glucosaminidase (NAG) converts the oligomers to monomers [44]. Two types of chitinase were expressed lowly in wildtype 932VR with no expression in *q-l*^*p*^ mutant. NAG (fold = 0.24) was also downregulated in *q-l*^*p*^. Downregulation of NAG and chitinase indicated the decrease of chitin degradation.

Increasing chitin biosynthesis and decreasing chitin metabolism might lead to accumulation of chitin. Correspondingly, many chitin-binding proteins were increased, consistent with the fact that more than 83.5% of differentially expressed cuticle protein genes were upregulated in *q-l*^*p*^. There were also more cuticle protein genes expressed in q-lp (96 genes expressed at RPKM≥5) than in 932VR (73 genes expressed at RPKM≥5). These results suggest that *q-l*^*p*^ requires more cuticle proteins than 932VR to produce its epidermis.

### Differentially expressed nuclear receptor genes

**Fig 6:**
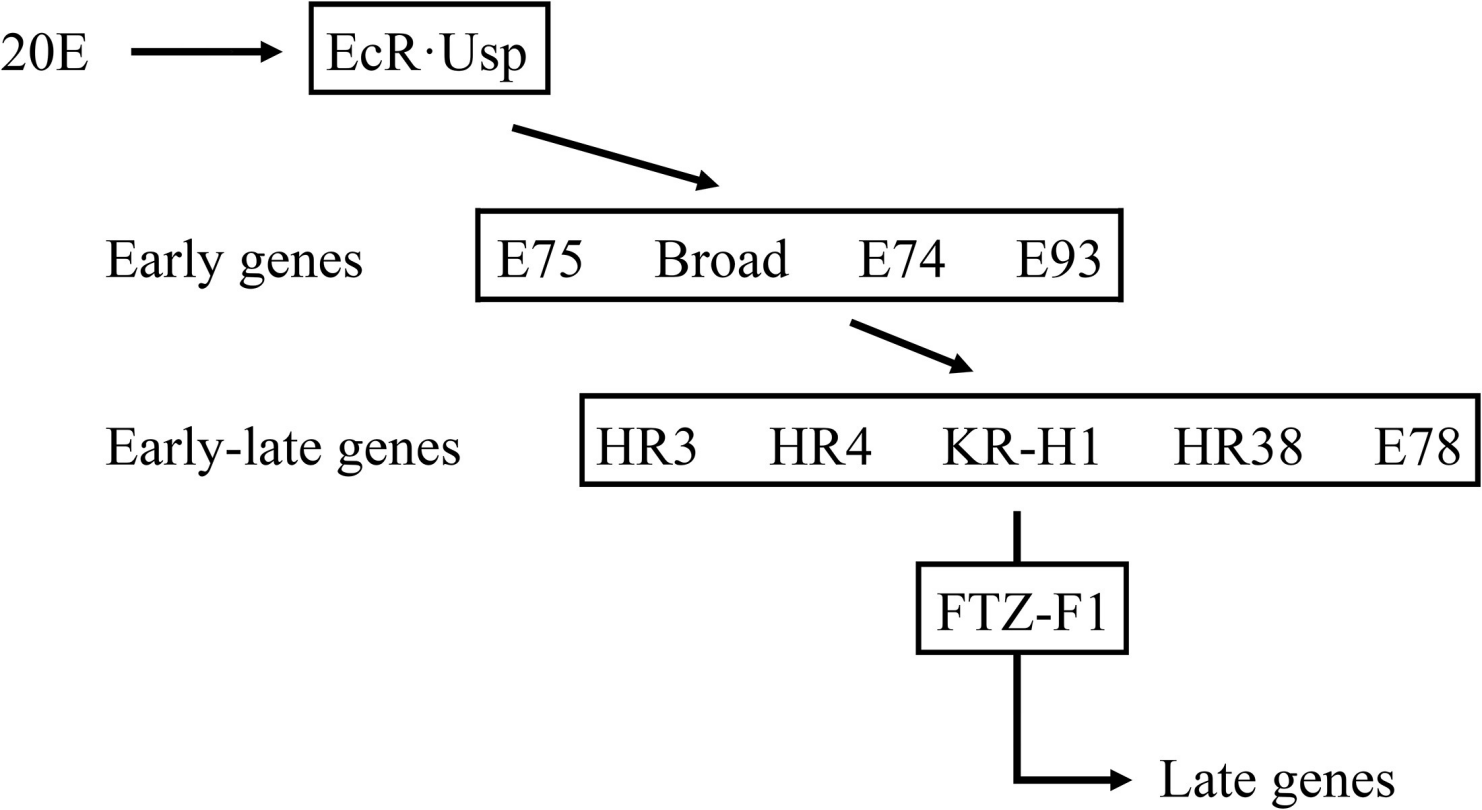
The signal transduction pathway of 20- hydroxyecdysone (20E).

As transcription factors and signal transduction-related genes were abundant among the differentially expressed genes, we examined several nuclear receptors. Nuclear receptors, a gene superfamily, interact with transcription factors to regulate the growth and development of eukaryotic cells [45]. There are two types of nuclear receptors, one of which has a known endogenous ligand; the other is termed an orphan receptor, as the ligand has yet to be identified [46]. In this study, we identified several common nuclear receptors, including FTZ-F1, E74a, HR38, HR39 and EcR [47], and the signal transduction pathway controlled by 20- hydroxyecdysone (20E) was exhibited in Fig 6. All of these receptors were upregulated, except for the ecdysone receptor (EcR, fold = 0.52).

HR38 belongs to the NR4A gene family, which can block the binding of heterologous dimers of EcR (ecdysone receptor) and USP (Ultra spiracle) [48, 49]. HR38 is also associated with epidermis formation [50, 51]. Upregulation of HR38 (fold = 2.51) might block the structure of EcR-USP heterologous dimers and affect the binding of EcR-USP and ecdysone [52], thus altering the downstream pathway regulated by ecdysone with correspondingly downregulation of the ecdysone receptor. Based on the above results, we hypothesized that upregulation of HR38 causes a reduction in EcR-USP and then blocks ecdysone signaling pathways, which might be responsible for developmental abnormalities phenotype, such as slow growth, low food consumption, and high mortality. Moreover, upregulation of HR38 also strengthens the synthesis of cuticle proteins, corresponding to the upregulated cuticular genes compared to downregulated cuticular genes in *q-l*^*p*^ and consistent with the finding that key enzymes of the chitin synthesis pathway were upregulated.

HR39 is necessary in female *D*. *melanogaster* flies due to its roles in copulation and fertilization. Additionally, HR39 also controls specific expression of cytochrome P450 [53, 54]. FTZ-F1 participates in larvae during molting and is associated with epidermization [55]. FTZ-F1 is highly homologous to HR39 [56], with the same binding sites in alcohol dehydrogenase (Adh), fushi tarazu (ftz), and new glue (ng) [57], indicating that FTZ-F1 and HR39 have other similar functions. E74a contains an E26 transformation-specific (ETS) domain and is induced by steroids to regulate transcription of genes such as L71-6 and L71-6, which are related to epidermis formation in pupae [58]. This steroid receptor was also upregulated in *q-l*^*p*^. Upregulation of these nuclear receptors is associated with epidermis formation, indicating that more cuticle proteins are synthesized in *q-l*^*p*^ than in 932VR. This was also consistent with the genes differentially expressed between 932VR and *q-l*^*p*^ and with the quantity and content of cuticle proteins in these strains.

## Conclusion

In this study, we analyzed the transcriptome of the epidermis silkworm (*B*. *mori*) *purple quail-like* mutant (*q-l*^*p*^) 4^th^-instar larvae during molting using RNA-Seq and validated the reliability by qRT-PCR. Our results allowed us to reach several conclusions. First, genes involved in biosynthesis of the pigments melanin, ommochrome and pteridine were all increased in the *q-l*^*p*^ mutant, which might be responsible for the pigmentation of the epidermis of this strain. In the melanin synthesis pathway, a large amount of dopamine melanin would be produced but less dopa melanin, NBAD sclerotin and NADA sclerotin. Second, a greater abundance of cuticle proteins were expressed in *q-l*^*p*^ than in 932VR, with significantly more upregulated than downregulated cuticular genes, indicating that the *q-l*^*p*^ mutant requires more cuticle proteins to produce its epidermis than 932VR. Third, the downstream pathway regulated by ecdysone appeared to be blocked by HR38 upregulation, and correspondingly we found that the ecdysone receptor was downregulated, which might be responsible for the developmental abnormalities phenotype of *q-l*^*p*^.

## Supporting information

S1 Table Primers for qRT-PCR and qRT-PCR and RPKM results. (XLSX)

S2 Table RPKMs of 14,623 known genes in *Bombyx mori q-l*^*p*^ and 932VR at 6 hours after molting of the 4^th^ instar. (XLSX)

S3 Table Differentially expressed genes between 932VR and *q-l*^*p*^. (XLSX)

S4 Table Expression of cuticle protein genes. (XLSX)
